# Supplementary material for: Nonlinear receptive fields evoke redundant retinal coding of natural scenes
Source: Nature. 2024 Nov 20;637(8045):394–401. doi: 10.1038/s41586-024-08212-3 (PMC11711096; doi:10.1038/s41586-024-08212-3)
Supplement: Supplementary file 1 — Reporting Summary [file 41586_2024_8212_MOESM1_ESM.pdf]

nature portfolio

Corresponding author(s): Tim Gollisch

Last updated by author(s): Sep 30, 2024

Reporting Summary

Nature Portfolio wishes to improve the reproducibility of the work that we publish. This form provides structure for consistency and transparency in reporting. For further information on Nature Portfolio policies, see our Editorial Policies and the Editorial Policy Checklist.

Statistics

For all statistical analyses, confirm that the following items are present in the figure legend, table legend, main text, or Methods section.

n/a	Confirmed	
☐	☒	The exact sample size (n) for each experimental group/condition, given as a discrete number and unit of measurement
☐	☒	A statement on whether measurements were taken from distinct samples or whether the same sample was measured repeatedly
☐	☒	The statistical test(s) used AND whether they are one- or two-sided<br>*Only common tests should be described solely by name; describe more complex techniques in the Methods section.*
☒	☐	A description of all covariates tested
☒	☐	A description of any assumptions or corrections, such as tests of normality and adjustment for multiple comparisons
☐	☒	A full description of the statistical parameters including central tendency (e.g. means) or other basic estimates (e.g. regression coefficient) AND variation (e.g. standard deviation) or associated estimates of uncertainty (e.g. confidence intervals)
☐	☒	For null hypothesis testing, the test statistic (e.g. F, t, r) with confidence intervals, effect sizes, degrees of freedom and P value noted<br>*Give P values as exact values whenever suitable.*
☒	☐	For Bayesian analysis, information on the choice of priors and Markov chain Monte Carlo settings
☒	☐	For hierarchical and complex designs, identification of the appropriate level for tests and full reporting of outcomes
☐	☒	Estimates of effect sizes (e.g. Cohen's d, Pearson's r), indicating how they were calculated

Our web collection on statistics for biologists contains articles on many of the points above.

Software and code

Policy information about availability of computer code

Data collection	Extracellular voltage signals were acquired with Multichannel Systems amplifiers. Visual stimuli were generated and controlled through custom-made software, based on Visual C++ and OpenGL. Raw data files and stimulus generation code can be made available upon reasonable request to the corresponding author.
Data analysis	Spike sorting was performed using a modified version of Kilosort (Pachitariu et al., 2016), available at https://github.com/dimokaramanlis/KiloSortMEA, and curated with the phy software (https://github.com/cortex-lab/phy). All analyses and generation of figures were done with MATLAB (versions 9.10 to 9.12). Code used to analyze spiking data and fit computational models is available on GitHub: https://github.com/dimokaramanlis/subunit_grid_model.

For manuscripts utilizing custom algorithms or software that are central to the research but not yet described in published literature, software must be made available to editors and reviewers. We strongly encourage code deposition in a community repository (e.g. GitHub). See the Nature Portfolio guidelines for submitting code & software for further information.

Data

Policy information about availability of data

All manuscripts must include a data availability statement. This statement should provide the following information, where applicable:

- Accession codes, unique identifiers, or web links for publicly available datasets
- A description of any restrictions on data availability
- For clinical datasets or third party data, please ensure that the statement adheres to our policy

All spike-sorted data used for this study are available at G-Node: https://doi.org/10.12751/g-node.ejk8kx (doi: 10.12751/g-node.ejk8kx). The applied natural images from the van Hateren database are available at https://pirsquared.org/research/vhatdb/full/.

Research involving human participants, their data, or biological material

Policy information about studies with human participants or human data. See also policy information about sex, gender (identity/presentation), and sexual orientation and race, ethnicity and racism.

Reporting on sex and gender	N/A
Reporting on race, ethnicity, or other socially relevant groupings	N/A
Population characteristics	N/A
Recruitment	N/A
Ethics oversight	N/A

Note that full information on the approval of the study protocol must also be provided in the manuscript.

Field-specific reporting

Please select the one below that is the best fit for your research. If you are not sure, read the appropriate sections before making your selection.

☒ Life sciences ☐ Behavioural & social sciences ☐ Ecological, evolutionary & environmental sciences

For a reference copy of the document with all sections, see nature.com/documents/nr-reporting-summary-flat.pdf

Life sciences study design

All studies must disclose on these points even when the disclosure is negative.

Sample size	No sample size calculations were used for this study. The sample size (here at least 3 retinas per species; each retina yields an unpredictable, but large number of recorded cells, typically several tens to hundreds) is consistent with the standards in the field for retinal multielectrode-array recordings and is comparable to sample sizes reported in similar publications (e.g., Roy et al 2021, Nature 492:409-413; Shah et al 2020, eLife 9:e45743). The retinal response properties investigated in this study were consistent across different recordings, and the sample size was sufficient to demonstrate the repeatability of the effects observed in both marmoset monkeys and mice.
Data exclusions	For all stimulus-specific population analyses, we excluded individual cells which did not reliably respond to the corresponding stimulus. Specific exclusion criteria are reported in the manuscript. For marmoset tissue, we only used retinas for which a 5% contrast full-field modulation at 4 Hz produced at least a 10 spikes/s modulation in the average ON parasol spike rate at the beginning of the recording.
Replication	All measurements in the study were performed on multiple cells of each type in multiple animals; cell numbers are reported in each of the relevant figure legends. There were no unsuccessful replication attempts; results were consistent across all recordings, and alls datasets that passed the reliability criterion stated above under Data exclusions were included in the final analysis.
Randomization	The study did not involve any traditional experimental groups and thus there was no requirement for randomization.
Blinding	The study did not involve traditional experimental groups that could be blinded. Data from all retinal ganglion cells were analyzed with the same code without selection. The researchers were blind to any group-dependent bias during data collection, because of the laborious offline analyses required for grouping cells into types.

Reporting for specific materials, systems and methods

We require information from authors about some types of materials, experimental systems and methods used in many studies. Here, indicate whether each material, system or method listed is relevant to your study. If you are not sure if a list item applies to your research, read the appropriate section before selecting a response.

Materials & experimental systems

n/a	Involved in the study
☒	☐ Antibodies
☒	☐ Eukaryotic cell lines
☒	☐ Palaeontology and archaeology
☐	☒ Animals and other organisms
☒	☐ Clinical data
☒	☐ Dual use research of concern
☒	☐ Plants

Methods

n/a	Involved in the study
☒	☐ ChIP-seq
☒	☐ Flow cytometry
☒	☐ MRI-based neuroimaging

Animals and other research organisms

Policy information about studies involving animals; ARRIVE guidelines recommended for reporting animal research, and Sex and Gender in Research

Laboratory animals	The study was performed with retinal tissue obtained from adult male marmoset monkeys (Callithrix jacchus), aged 12-18 years, and wild-type female mice (C57BL/6J), aged 7-23 weeks. Mice were housed in a 12-hour light/dark cycle.The ambient conditions in the animal housing room were kept at around 21°C (20–24°C) temperature and near 50% (45–65%) humidity.
Wild animals	The study did not involve wild animals.
Reporting on sex	The study did not involve sex-based analyses, because previous literature suggests that the electrophysiological properties of retinal tissue is relatively homogeneous between sexes.
Field-collected samples	The study did not involve collection of samples from the field.
Ethics oversight	Experimental procedures were in accordance with national and institutional guidelines and approved by the institutional animal care committee of the University Medical Center Göttingen, the German Primate Center and by the responsible regional government office (Niedersächsisches Landesamt für Verbraucherschutz und Lebensmittelsicherheit, permit number 33.19-42502-04-20/3458).

Note that full information on the approval of the study protocol must also be provided in the manuscript.
